# Mapping Alzheimer’s disease: exploring cellular vulnerability and resilience

**DOI:** 10.1038/s41392-024-02014-9

**Published:** 2024-10-28

**Authors:** Natalia Ortí-Casañ, John R. Bethea, Ulrich L. M. Eisel

**Affiliations:** 1 Department of Molecular Neurobiology, Groningen Institute for Evolutionary Life Sciences, University of Groningen, Groningen, The Netherlands; 2 Department of Anatomy and Cell Biology, The George Washington University School of Medicine and Health Sciences, Ross Hall, Washington, DC USA

**Keywords:** Molecular neuroscience, Neurology

In a recent paper published in Nature, Mathys and co-workers presented a comprehensive and precise transcriptomic atlas of distinct brain regions from individuals with and without Alzheimer’s disease (AD). This paper aimed to identify differences in regional molecular architecture along with region-specific changes in neuronal and glial subtypes, while also investigating whether certain brain regions and cell subtypes are more vulnerable or uniquely affected by AD compared to others, and exploring mechanisms that may contribute to cellular resilience.^1^

Alzheimer’s disease (AD) is a progressive neurodegenerative disease characterized by cognitive decline and structural brain changes. Its key pathological features are extracellular amyloid-β plaques and intracellular neurofibrillary tangles (NFTs), each following a specific brain distribution pattern. In early stages, tangles are thought to be confined to the entorhinal cortex and hippocampus (Braak stages I–III), regions critical for memory. As the disease progresses, tangles spread to the neocortex (Braak stages IV–VI), correlating with worsening cognitive impairment.^2^ Despite decades of research, the precise cellular and molecular mechanisms driving the onset and progression of AD remain elusive making it one of the most challenging neurodegenerative disorders of our time. Previous studies have focused on individual brain regions but a detailed region-specific molecular characterization of the human brain in the context of AD is lacking.

In the study by Mathys et al., an initial examination performed by single-nucleus RNA sequencing (snRNA-seq) revealed differences in the composition of major cell types, namely neurons and glial cells, across various brain regions. These patterns were consistent across individuals regardless of AD status, indicating that this variability is an integral aspect of the human brain. The construction of the brain atlas of AD-affected brain regions revealed that excitatory neurons showed strong regional specificity in areas like the hippocampus and thalamus, while inhibitory neurons were broadly distributed across cortical regions. Regarding glial cells, a novel technique, single cell decorrelated module networks (scdemon), revealed that, in contrast to other immune cells, astrocytes showed high regional heterogeneity and expressed gene programs associated to synapse interaction and other functions. Overall, this atlas has provided a deeper understanding of cellular architecture and signaling mechanisms that impact AD. Although the study covers six distinct brain regions, inclusion of other areas, such as the cerebellum or brain stem, could be relevant for AD pathology, as these areas may play roles in early or atypical manifestations of the disease. Furthermore, while the study profiled over 1.3 million nuclei from 283 post-mortem human brain samples, these were obtained from only 48 participants, which still represents a relatively small cohort. Moreover, the samples were obtained from one single ROSMAP cohort. Therefore, larger and more diverse sample sizes could provide more valuable findings, especially considering the heterogeneity of AD pathology and progression.

**Fig. 1:**
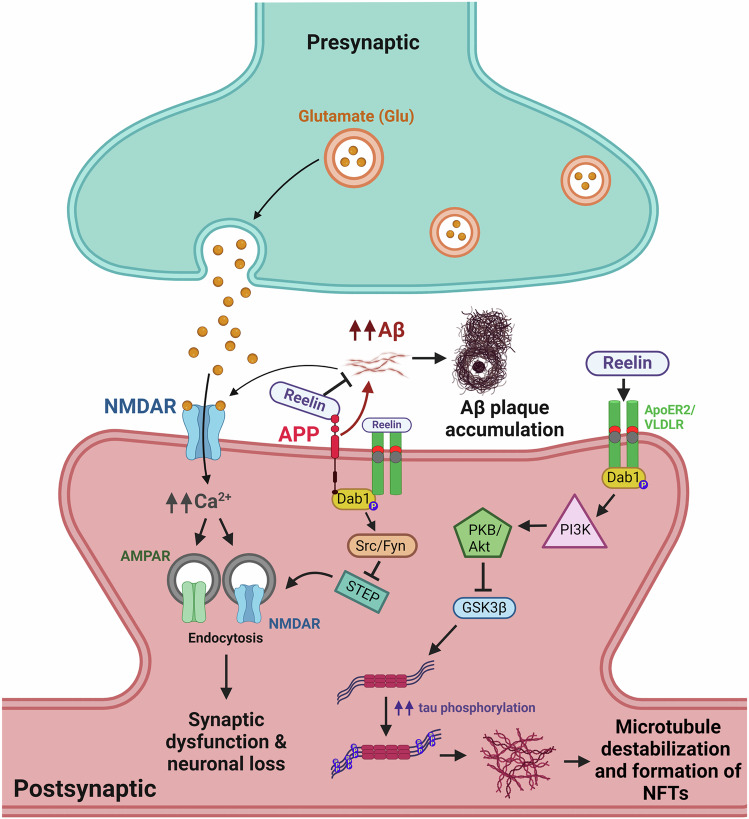
Reelin signaling pathway in Alzheimer’s disease (AD). During healthy conditions, Reelin signaling prevents abnormal tau phosphorylation, modulates amyloid precursor protein (APP) processing and enhances NMDA and AMPA receptors functions. Reelin binds to its receptors very-low-density lipoprotein receptor (VLDLR) and apolipoprotein E receptor 2 (ApoER2). This binding activates the adaptor protein Disabled-1 (Dab1) through phosphorylation, which initiates downstream pathways, including activation of phosphatydilinositol-3-kinase (PI3K) and protein kinase B (PKB/Akt), involved in synaptic plasticity, cytoskeletal stability and neuronal maintenance, among others.^3^ Reelin is involved in the downregulation of tau phosphorylation via reduction of Glycogen synthase kinase-3 beta (GSK3β) activity. However, alterations in Reelin expression during AD results in the reduced activation of its downstream signaling, leading to excessive tau phosphorylation, microtubule destabilization and formation of NFTs. Moreover, Reelin can interact with APP and promote its non-amyloidogenic processing. Thus, altered Reelin signaling leads to decreased inhibition of amyloidogenic APP processing, contributing to increased Aβ production and Aβ plaque accumulation.^3^ Finally, Reelin signaling can activate Src/Fyn kinases, leading to the inhibition of striatal-enriched protein tyrosine phosphatase (STEP), which in turn prevents NMDA receptor endocytosis. In fact, during AD, activation of STEP by Aβ leads to internalization of NMDA receptors.^3^ Hence, impaired Reelin signaling causes the dysregulation of NMDA receptor activity, leading to the weakening of synaptic strength and reduced trafficking of AMPA receptors. Additionally, Aβ can also bind NMDA receptors, leading to its overstimulation and excessive release of Ca^2+^, which causes the endocytosis of both AMPA and NMDA receptors.^3^ Figure created with BioRender.com

One of the most striking findings of this study was the identification of cell type-specific vulnerabilities in AD, notably the significant depletion of a specific subtype of excitatory neurons, which was linked to poorer cognitive performance. This vulnerable subtype was associated with the Reelin signaling pathway, a key regulator of synaptic plasticity and neuronal migration. It has been demonstrated that, in the context of AD, reduced Reelin expression is associated with increased tau phosphorylation, amyloid plaque formation and synaptic dysfunction^3^ (Fig. 1). Indeed, human and animal studies confirmed that Reelin-expressing neurons are particularly vulnerable in AD.^3^ Thus, the discovery of the potential role of Reelin in contributing to neuronal vulnerability makes it an attractive target for therapeutic intervention. Further studies on Reelin signaling are needed to clarify its precise role in Alzheimer’s disease. Mechanistic experiments in animal models, such as the Reelin-COLBOS mouse model,^4^ could help identify the specific pathways contributing to cellular vulnerability and validate findings from post-mortem research.

Furthermore, the study identified differentially expressed genes (DEGs) across different brain regions and cell types to investigate cellular and functional differences in AD. Notably, microglia and immune cells showed strong associations with AD-related genes based on genome-wide association studies in specific brain areas, such as hippocampus and thalamus. Additionally, when exploring DEGs associated with AD pathology, i.e., NFTs and amyloid-β plaques, a significant overlap was observed in the entorhinal cortex and hippocampus while less DEGs overlapped in the prefrontal cortex and angular gyrus. These regional differences coincide with AD progression, where NFTs appear later in neocortical regions. These findings highlight distinct molecular pathways for NFTs and plaques, potentially leading to targeted therapies. To enhance our knowledge about the progression of AD pathology, future studies could focus on implementing a network-based approach to examine the interactions between different brain regions rather than studying them separately. Understanding how these regions communicate, and function dynamically is crucial for a comprehensive view of how the disease spreads.

The analysis also revealed important changes associated with glial cells. Astrocytes exhibited more plaque-associated DEGs than any other cell type and all glial cells showed an up-regulation of glycolysis-related genes in response to AD pathology, indicating specific metabolic pathways disrupted in AD. The discovery of the key alterations that the immune components of the brain undergo during AD highlight the necessity of further investigating the immune system and neuroinflammation as key players in disease progression in AD. Indeed, multiple studies have demonstrated that targeting the immune system is effective in restoring AD-related neuropathology.^5^

This study also investigated transcriptional changes related to cognitive resilience in AD. Cognitive resilience is referred to how particular individuals are able to retain a better cognitive function despite presenting significant AD pathology. By examining DEGs in various cell types, the analysis identified astrocytes as critical players in mediating cognitive resilience in AD. This analysis revealed key molecular pathways including antioxidant activity, choline metabolism and polyamine biosynthesis, offering new potential therapeutic approaches for enhancing cognitive resilience in AD.

This paper offers valuable insights but also has some limitations. First, the relatively small sample size used, which belongs to one single ROSMAP cohort restricts the generalizability of the findings. A follow-up study using multi-institutional cohorts to validate the reported findings could be performed. Furthermore, the findings of the study were based on post-mortem brain tissue at a single time point. A more longitudinal design could help better understand how cellular changes evolve over time as AD pathology progresses. For instance, measuring cerebrospinal (CSF) samples could highlight certain molecular brain changes over time. A more in-depth analysis of molecular mechanisms as well as sex-dimorphic clusters is lacking in the study. Thus, more functional studies employing animal models or induced pluripotent stem cells (iPSC) might complement the study’s findings. Moreover, a clearer correlation between the molecular findings and the clinical phenotypes would have facilitated the interpretation of findings regarding cellular vulnerability and resilience. Finally, though the study employs advanced transcriptomic techniques, potential limitations in single-cell analysis or biases may lead to underestimations of certain pathways or genes.

In conclusion, this paper represents a significant advancement in the understanding of AD by providing a detailed region-specific molecular atlas of the human AD brain. Additionally, making this data available on an online platform further facilitates its accessibility for the scientific community.
